# Summer Course of Medical Instruction

**Published:** 1859-02

**Authors:** 


SUMMER COURSE OF MEDICAL INSTRUCTION.

It will be seen by the following circular, that the Faculty of Rush Medical College will continue a valuable course of medical instruction through the coming spring and first part of summer.

Good board and rooms can be obtained for from $3 to $3.50 per week; and all such students as can meet the required expenses will find it a great advantage to spend the time proposed in this city. The circular is as follows:

RUSH MEDICAL COLLEGE. — SUMMER COURSE OF LECTURES.—

| | |
|---|---|
| *Clinical Surgery*, . . . . . . | D. Brainard, M.D. |
| *Clinical Medicine*, . . . . . . | N. S. Davis, " |
| *General Anatomy*, . . . . . . . | J. W. Freer, " |
| *Diseases of Women and Children*, . | W. H. Byford, " |
| *Botany and Vegetable Physiology*, . | John H. Rauch, " |

*Prof. Brainard* will lecture at the Marine Hospital and at the College Dispensary. He will also examine Students on Surgery, and require them to prepare Essays and report Cases.

*Prof. Davis* will lecture at the Mercy Hospital. Special attention will be paid to Auscultation and Percussion, Microscopic and Chemical Examinations of Morbid Products. He will also examine Students on the Practice of Medicine, and require them to prepare Essays and report Cases.

*Prof. Freer* will lecture at the College, and illustrate his Course as far as practicable by the Microscope. He will also examine Students on Anatomy.

*Prof. Byford* will lecture at the College. His connection with the College Dispensary will enable him to illustrate much of his Course by Clinical Cases. He will also examine Students on Obstetrics, and require them to prepare Essays.

*Prof. Rauch* will lecture at the College. Students will have access to his Collection and Herbarium, and as soon as vegetation is sufficiently advanced, he will take them into the field. He will also examine Students on Materia Medica, and require them to prepare Essays.

The Course begins Monday, April 3d, and continues until July 16th. Two lectures a day will be delivered. It will be seen that the object of the Course is to teach practically as much as possible, and also to teach subjects which, owing to a want of time, are either partially or entirely excluded from the Winter Course.

Fee for each Professor, Ten Dollars. Students can take one or all the tickets.

